# Registry of Compartmental Ephrin-B3 Guidance Patterns With Respect to Emerging Multimodal Midbrain Maps

**DOI:** 10.3389/fnana.2021.649478

**Published:** 2021-03-16

**Authors:** Jeremiah P. C. Stinson, Cooper A. Brett, Julianne B. Carroll, Mark L. Gabriele

**Affiliations:** Department of Biology, James Madison University, Harrisonburg, VA, United States

**Keywords:** inferior colliculus, multisensory, receptor tyrosine kinases (RTKs), GAD, transgenic, calretinin, mosaic, modularity

## Abstract

Guidance errors and unrefined neural map configurations appear linked to certain neurodevelopmental conditions, including autism spectrum disorders. Deficits in specific multisensory tasks that require midbrain processing are highly predictive of cognitive and behavioral phenotypes associated with such syndromes. The lateral cortex of the inferior colliculus (LCIC) is a shell region of the mesencephalon that integrates converging information from multiple levels and modalities. Mature LCIC sensory maps are discretely-organized, mimicking its compartmental micro-organization. Intermittent modular domains receive patchy somatosensory connections, while inputs of auditory origin terminate in the encompassing extramodular matrix.Eph-ephrin signaling mechanisms instruct comparable topographic arrangements in a variety of other systems. Whether Eph-ephrin interactions also govern the assembly of LCIC multimodal maps remains unaddressed. Previously, we identified EphA4 and ephrin-B2 as key mediators, with overlapping expression patterns that align with emerging LCIC modules. Here, we implicate another member of this guidance family, ephrin-B3, and quantify its transient expression with respect to neurochemically-defined LCIC compartments. Multiple-labeling studies in GAD67-GFP knock-in mice reveal extramodular ephrin-B3 expression, complementary to that of EphA4 and ephrin-B2. This distinctive pattern sharpens over the early postnatal period (birth to P8), prior to ephrin-B3 downregulation once multimodal LCIC inputs are largely segregated (P12). Channel-specific sampling of LCIC ROIs show ephrin-B3 signal periodicities that are out-of-phase with glutamic acid decarboxylase (GAD;modular marker) signal fluctuations, and match calretinin (CR) waveforms (matrix marker). Taken together, the guidance mosaic registry with emerging LCIC compartments and its interfacing afferent streams suggest a prominent role for Eph-ephrins in ordering behaviorally significant multisensory midbrain networks.

## Introduction

One important function of the midbrain is the integration of multisensory cues critical for reflexive and orientation behaviors (Gruters and Groh, 2012; Louthan et al., 2020). Such processing requires highly organized circuits that are thought to develop *via* both activity-dependent and activity-independent mechanisms (Cang and Feldheim, 2013; Cramer and Gabriele, 2014). Precise alignment of converging multimodal maps in deep aspects of the superior colliculus (SC) is well documented (Triplett et al., 2012), with activity driving the registry of auditory (King et al., 1988; Knudsen and Brainard, 1991) and visual cortical maps for retinocollicular inputs, while topographic projections from primary somatosensory cortex rely upon positional cues provided by complementary ephrin-A/EphA guidance gradients (Rashid et al., 2005; Miller et al., 2006).

The lateral cortex of the inferior colliculus (LCIC) is situated just downstream of the SC and receives an equally impressive array of multisensory inputs. In addition to passing on auditory space-mapped representations through topographic projections to the SC (Knudsen and Knudsen, 1983), lesioning studies of the LCIC suggest a critical role as an inhibitory modulator of the acoustic startle response (ASR; Parham and Willott, 1990). Altered startle responses and impaired reflex modifications (e.g., pre-pulse inhibition) are highly predictive of subsequent speech and language dysfunction in humans and certain neurodevelopmental disorders (Kwakye et al., 2011; Stevenson et al., 2014a, b). Abnormalities in multisensory map formation, alignment, and subsequent pruning events are thought in part to potentially underlie such conditions (Wurzman et al., 2015).

Compared to the SC, very little is known about the mechanisms involved in LCIC circuit assembly. Modality-specific inputs to the LCIC are segregated in the adult mouse (Lesicko et al., 2016), terminating within discrete zones along its neurochemically-defined compartmental framework (Chernock et al., 2004). Somatosensory afferents exhibit discontinuous patchy distributions, targeting layer 2 modular fields positive for glutamic acid decarboxylase (GAD). Auditory afferent patterns are complementary in nature, ending in the surrounding matrix or extramodular domains. Local LCIC processing flows largely unidirectionally, from the matrix to modules (Lesicko et al., 2020). LCIC efferent systems also appear to be compartmentally organized, with the extramodular matrix projecting bilaterally to other IC subdivisions and the SC, while modular domains, in turn, target the medial division of the medial geniculate body and the closely associated posterior limitans and posterior intralaminar nuclei. Such an arrangement allows for routing of matrix auditory processing to appropriate midbrain targets, while also providing a means for multisensory integration within the LCIC (i.e., modules), before its transmission on to higher multimodal centers. Seeing as identified modular targets are interconnected with the amygdala and other limbic structures, it is plausible such pathways provide the multimodal context necessary for executing conditioned fear behaviors (Ledoux et al., 1987).

Previous work from our lab characterized the emergence of LCIC compartments and identified calretinin (CR) as a reliable extramodular marker during an early critical period (Dillingham et al., 2017). The mechanisms responsible for the development of the distinctive LCIC mosaic and its interfacing afferent-efferent systems remain largely unexplored. Recently, we implicated two members of the Eph-ephrin guidance family as playing a potential role. EphA4 and ephrin-B2 are transiently expressed in the nascent LCIC in overlapping patterns that align with its GAD-positive modular zones (Gay et al., 2018). Concurrently, major afferent patterns are being shaped, targeting discrete LCIC zones in a modality-specific manner that appears adult-like by postnatal day 12 (Lamb-Echegaray et al., 2019). The present study utilizes multiple-labeling approaches in a GAD67-GFP knock-in mouse line to evaluate another member of this signaling family, ephrin-B3. Here, we show a temporally matched, albeit complementary LCIC matrix expression pattern for ephrin-B3. Potential Eph-ephrin influences in establishing multisensory LCIC circuits, as well as their role in instructing contrasting neural map features (continuous vs discrete) in neighboring IC subdivisions during an early critical period are discussed.

## Materials and Methods

### Animals

Experiments were performed in C57BL/6J (*n* = 4) and heterozygous GAD67-GFP transgenic mice (*n* = 38) over a defined critical period (birth, postnatal day 0, P0, P4, P8, and P12, *n* ≥ 4 at each age) for LCIC compartmentalization and developing afferent patterns (Dillingham et al., 2017; Gay et al., 2018; Lamb-Echegaray et al., 2019). An approximately equal number of males and females were used, with no observed gender-specific differences. C57BL/6J mice were used in pilot studies to verify antibody specificity and to establish optimal working dilutions in neonatal tissue (*data not shown*). Specifics concerning the generation of the GAD67-GFP (Δ neo) line are described elsewhere (Tamamaki et al., 2003; permission granted by Dr. Yuchio Yanagawa, Gunma University Graduate School of Medicine, Gunma Japan). We previously validated this line in the neonate showing GFP colocalization with GAD67 immunolabeling across IC subdivisions (Gay et al., 2018). All procedures were performed in keeping with the National Institutes of Health *Guide for the Care and Use of Laboratory Animals* and received prior approval by the Institutional Animal Care and Use Committee at James Madison University (Protocol No. 20-1421).

### Immunohistochemistry

A coronal series of midbrain sections at each of the designated ages was collected and processed similar to that previously reported in detail (Dillingham et al., 2017; Gay et al., 2018; Lamb-Echegaray et al., 2019). Briefly, brains were fixed transcardially with 4% paraformaldehyde (pH 7.4) and subsequently cryoprotected at 4°C in increasing concentrations of sucrose solution (10–30%) in the same fixative. Sections were cut at a thickness of 50 μm on a sliding freezing microtome and collected in 0.1 M phosphate-buffered saline (PBS, pH 7.4). Initial PBS rinses (3 × 10 min each) were performed on free-floating sections before a blocking step in either 5% normal donkey or horse serum (defined by secondary antibody species) for 30 min. Tissue was then incubated in primary antibody (Supplementary Table 1: anti-CR, made in rabbit, 1:250, CR 7697, Swant, RRID: AB_2619710; anti-ephrin-B3, made in goat, 1:200, AF395, R&D Systems, RRID: AB_2095814) at room temperature for 40 min and agitated overnight at 4°C. For double-labeling studies, a directly conjugated secondary was used for CR visualization (Alexa Fluor 350 donkey anti-rabbit IgG, 1:25, A10039, Thermo Fisher Scientific, RRID: AB_2534015), paired with a biotinylated-streptavidin detection system for ephrin-B3 (biotinylated horse anti-goat IgG, 1:600, BA-9500, Vector Laboratories, RRID: AB_2336123; Dylight 549 streptavidin, 1:200, SA-5549, Vector Laboratories). Following final PBS rinses, sections were mounted in serial order on charged slides and coverslipped with ProLong Diamond anti-fade protection (P-36970, Thermo Fisher Scientific).

### Imaging, LCIC Sampling, and Statistical Analyses

A Nikon TE2000 microscope (Nikon, Melville, NY, USA) fitted with a Hamamatsu ORCA-Flash 4.0 V3 sCMOS monochrome camera (Hamamatsu, Bridgewater, NJ, USA) was used for epifluorescence capturing of individual channels throughout the rostrocaudal extent of the LCIC. Filter sets (Chroma Technology, Bellows Falls, VT, USA) designed specifically to prevent bleed-through enabled unequivocal identification of the various fluorophores. Acquired Z-stacks for each channel were pseudocolored (red: ephrin-B3, green: GAD, blue: CR) and flattened using an extended depth of field (EDF) deconvolution algorithm (Elements Software; Nikon).

Individual channels for representative sections through aspects of the LCIC where its modular-extramodular organization is most evident were extracted separately as uncompressed TIFFS, opened in ImageJ (NIH, Bethesda, MD, USA), and converted to grayscale for pattern sampling and generation of brightness profile data sets for quantitative assessments. A freehand tool with a line thickness of 100 (±25 depending on developmental stage) was used to sample LCIC layer 2, bisecting discontinuous GAD-positive modules. Sampling contours were drawn initially on imported GAD channels in a ventral-to-dorsal progression. The region of interest (ROI) manager was used in ImageJ to replicate the identical contour drawn in the GAD channel for each subsequent channel sampled. LCIC layer 2 sampling was similarly performed in P0 mice, although GAD-positive modules were not readily apparent at this earliest time point (Dillingham et al., 2017; Gay et al., 2018; Lamb-Echegaray et al., 2019). As LCIC ephrin-B3 expression was sharply downregulated and negligible by P12, no sampling or quantitative analyses were performed at this age. Generated series data were graphed together as brightness plot profiles to qualitatively assess relative signal matching/mismatching. Raw data values for each channel were exported for further quantitative measures described below.

Signal processing was performed in keeping with previous protocols assessing continuous and discrete IC developmental patterns (Fathke and Gabriele, 2009; Gabriele et al., 2011; Wallace et al., 2013, 2016; Dillingham et al., 2017; Gay et al., 2018; Lamb-Echegaray et al., 2019). Cross-correlation is a well-established signal processing technique to assess the similarity of two signals or series. In brief, it displaces the two at varying shifts with respect to each other to numerically quantify similar pattern features. Cross-correlation analyses were performed in Microsoft Excel (Redmond, WA) using the CORREL function, quantifying the relative spatial registry of ROI signal pairings (ephrin-B3 and GAD) at each age with detectable ephrin-B3 expression (P0, P4, P8). The resulting cross-correlation function y-intercept values (the origin represents no relative signal shift) provided a metric for the spatial overlap of the two channels at each of the examined stages. Cross-correlation function values span from +1.0 to −1.0. Higher values near +1.0 indicate strong pattern alignment or overlapping signal fluctuations. In contrast, more negative values indicate complementary patterns or signals increasingly out-of-phase with each other. Values near zero suggest no relative signal variations. A minimum of four sections (*n* ≥ 4) was analyzed at each age and included in the statistical analyses. Age differences in this metric were tested using independent, two-tailed Student’s *t*-tests assuming unequal variance (Type 3) with statistical significance (*p* < 0.05).

## Results

### Transient Ephrin-B3 Expression Complementary to GAD-Defined Modules

**Figure 1:**
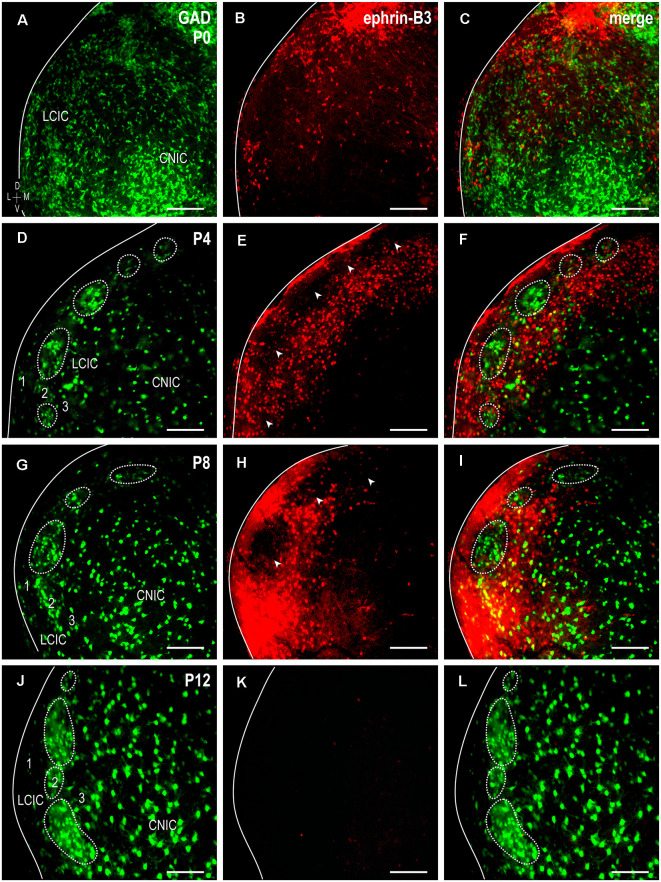
Developmental progression of GAD67-GFP and ephrin-B3 expression patterns in the neonatal lateral cortex of the inferior colliculus (LCIC). Transient ephrin-B3 expression (red) with respect to developing glutamic acid decarboxylase (GAD)-positive modules (green) at P0 **(A–C)**, P4 **(D–F)**, P8 **(G–I)**, and P12 **(J–L)**. At birth, GAD-positive modules are in early development and are not readily identifiable, and ephrin-B3, while present in the LCIC, does not yet exhibit any clear compartmental organization. By P4, modules are distinguishable (dashed contours) and become more evident with age. Over this critical period for emerging LCIC compartments, the ephrin-B3 expression is complementary to that of GAD and preferentially restricted to the surrounding LCIC matrix. Note discrete layer 2 modular voids at P4 and P8 (**E,H**, arrowheads), encompassed by concentrated ephrin-B3 expression. With the modular-extramodular framework and afferent patterns largely in place by P12 (*see* Lamb-Echegaray et al., 2019), LCIC ephrin-B3 expression appears strikingly downregulated **(K,L)**. Scale bars = 100 μm.

To assess ephrin-B3 expression patterns with respect to the developing LCIC modularity, immunostaining was performed in GAD67-GFP mice at the four designated stages spanning the early critical period (Figures 1A–L). As previously reported (Dillingham et al., 2017; Gay et al., 2018; Lamb-Echegaray et al., 2019), a discontinuous series of GAD-positive modules is not yet clearly discernable at birth (Figure 1A). Ephrin-B3 is present at P0, largely localized to the LCIC, and most heavily concentrated in dorsal aspects (Figures 1B,C). By P4, characteristic GABAergic modules dominate layer 2 (Figure 1D, dashed contours), with ephrin-B3 expression further restricted to the LCIC and more uniformly distributed than that observed at birth. Despite this seemingly more even ventrodorsal expression, periodic voids of labeling are encountered along this dimension in layer 2 (Figure 1E, arrowheads). Encompassing ephrin-B3 expression occupies layers 1 and 3, as well as intermodular spaces, exhibiting characteristic extramodular patterns complementary to GAD-defined modular fields (Figure 1F). LCIC ephrin-B3 expression peaks at P8, maintaining its matrix-like surround and non-overlap with GAD-positive modules (Figures 1G–I). This robust expression is downregulated by P12, as ephrin-B3 is undetectable in the LCIC at this age (Figures 1J–L).

### Quantitative Assessment of Guidance Patterns With Respect to Emerging LCIC Compartments

**Figure 2:**
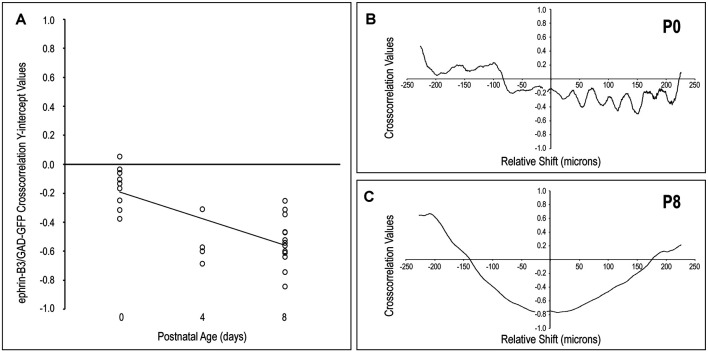
Quantification of LCIC ephrin-B3 expression patterns relative to GAD modular expression over the early critical period. The negatively sloped linear regression of cross-correlation y-intercept values with age **(A)** supports the observed developmental trend that ephrin-B3 patterns become increasingly extramodular and offset from emerging GAD-positive layer 2 modules. The absence of any prominent values in P0 cross-correlation functions [representative plot shown in panel **(B)**] indicates the lack of any significantly similar pattern features between ephrin-B3 and GAD-positive signals at birth. In contrast, by P8 [representative plot shown in panel **(C)**] pronounced negative cross-correlation y-intercepts and smooth transitions to prominent positive cross-correlation values at shifts of approximately ±200–250 μm indicate a significant mismatch between signal features at zero relative signal shift and again at regular intervals in keeping with the discontinuous LCIC compartmental arrangement at this age.

In keeping with qualitative observations, ephrin-B3 expression patterns and GAD-positive modular domains become increasingly distinct from birth to P8, as shown by cross-correlation analyses (Figure 2). No cross-correlation analyses were performed at P12 given the transience of ephrin-B3 expression and the absence of any LCIC labeling at this latest time point. Y-intercept values at all examined ages are either essentially zero (i.e., no correlation between signals) or consistently negative (i.e., indicative of signal offset, Figure 2A). The lack of any remarkably positive values at any age underscores the non-overlapping nature of these two signals. At birth, y-intercept values are reliably clustered about zero (suggesting no detectable correlation) or slightly negative (suggesting hints of early pattern separation). By P4 and P8, y-intercept values are collectively more negative, indicating increasing mismatch or out-of-phase signal fluctuations. A negatively sloped linear regression supports the observed developmental trend that ephrin-B3 patterns become increasingly aligned with the surrounding matrix with age, and offset from emerging GAD-positive layer 2 modules. Furthermore, comparisons of cross-correlation y-intercept values are statistically significant for P0 vs P4 and P0 vs P8 (*p* < 0.05 for both; *p* = 0.035 and *p* < 0.001, respectively), but not P4 vs. P8 (*p* > 0.05; *p* = 0.919).

Off-origin cross-correlation function peaks and troughs indicate matching and mismatching signal variations, respectively, occurring at particular relative spatial shifts between the two signals. At birth, y-intercepts (i.e., no relative shift) are close to zero, while non-zero signal shifts produce values that oscillate arbitrarily at brief intervals (every 25–50 μm) that do not reflect any anatomically significant features regarding the developing LCIC architecture (Figure 2B). In contrast, P8 cross-correlation functions exhibit distinctly negative y-intercepts and prominent off-origin maxima (Figure 2C), that approximate known periodicity regarding neuroanatomical LCIC compartments at this age (Dillingham et al., 2017; Gay et al., 2018; Lamb-Echegaray et al., 2019). Taken together, these quantitative measures support the notion of sharpening ephrin-B3 expression patterns over this early critical period that become increasingly mismatched with emerging LCIC modular domains.

### Ephrin-B3 Expression Overlaps CR-Defined Matrix

**Figure 3:**
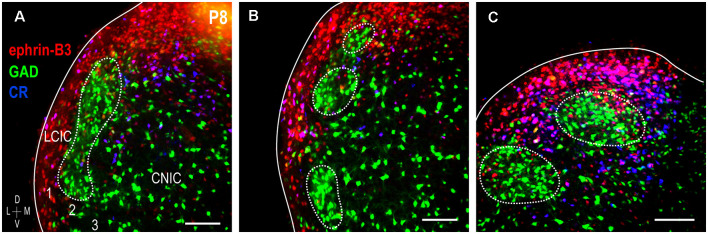
Ephrin-B3 LCIC expression with respect to GAD (modular) and calretinin (CR; matrix) labeling at P8. Digital merges from a coronal series **(A–C)** through the modular extent of the LCIC at P8 [ephrin-B3 = red, GAD = green, calretinin (CR) = blue]. Dashed contours indicate the change in the appearance of GAD modules along the caudal-to-rostral dimension of the LCIC (in keeping with that previously described, see Dillingham et al., 2017; Gay et al., 2018). The ephrin-B3 expression is strong throughout the coronal series, exhibiting patterns complementary to GAD-positive modules that overlap the calretinin-defined matrix. Within layers 1 and 3 and intermodular bridges that together comprise extramodular regions, many calretinin-positive cells are also positive for ephrin-B3 (purple). Scale bars = 100 μm.

Double-labeling experiments for ephrin-B3 and CR (an established extramodular marker) were performed in P8 GAD67-GFP mice to further confirm its matrix expression pattern. This age was chosen as it is the height of observed ephrin-B3 expression and pattern specificity, before its downregulation at P12. Both ephrin-B3 and CR are expressed throughout the LCIC extent that GAD-GFP modules span (Figure 3). Consistent throughout this expanse is their co-localization within areas that encompass GAD-defined zones. Layer 2 GAD-positive fields are interconnected caudally and more characteristically patchy within mid- and rostral regions (Figures 3A–C, dashed contours). Regardless of the coronal level and modular appearance, ephrin-B3, and CR distributions are matching and reliably complementary to that of GAD. Ephrin-B3 and CR-positive cells are abundant throughout the extramodular expanse and rarely encountered within modular confines. At each LCIC level, both single- (ephrin-B3: red, CR: blue) and double-labeled (ephrin-B3 and CR: purple) neurons are prevalent within extramodular zones.

**Figure 4:**
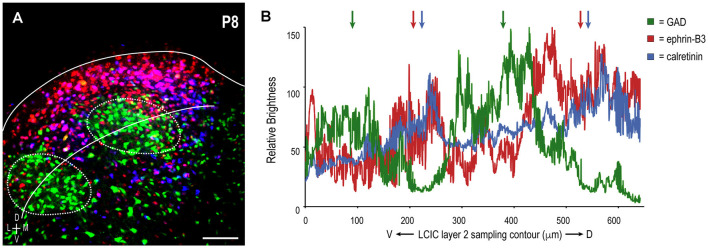
LCIC sampling and corresponding brightness profiles of extracted GAD (green), ephrin-B3 (red), and calretinin (blue) channels in a representative P8 mouse. Consistent ROI sampling curved contour in panel **(A)** bisecting LCIC layer 2 modular fields facilitates comparisons of relative signal fluctuations for each label **(B)**. Each exhibits a periodic component, in keeping with discontinuous compartments along the ventrolateral-to-dorsomedial extent of the LCIC. Ephrin-B3 and calretinin waveforms are largely in-phase with each other (red and blue arrows, respectively), and out-of-phase with that of GAD oscillations (green arrows). Such reliable alignment (with CR) and offset (with GAD) suggests a high degree of ephrin-B3 expression specificity that corresponds with the emerging LCIC extramodular matrix. Scale bar in **(A)** = 100 μm.

A sampling of multiple labels at P8 reveals three periodic channel waveforms with combinatorial readouts that were either in- or out-of-phase with each other (Figure 4A). GAD waveform oscillations are consistently offset spatially from those for ephrin-B3 and CR, which appear remarkably similar (Figure 4B), colored arrows). In sum, ephrin-B3 and CR patterns are overlapping and complementary to GAD, establishing ephrin-B3 as a temporally- and spatially-regulated guidance molecule that is associated with the emerging LCIC matrix.

## Discussion

Nuclear compartmentalization and guidance of specific inputs to emerging organizational features are critical steps in the establishment of highly integrative networks. The LCIC epitomizes such an orderly arrangement, with its modular-extramodular framework and converging inputs of multiple modalities (Chernock et al., 2004; Lesicko et al., 2016; Dillingham et al., 2017; Gay et al., 2018; Lamb-Echegaray et al., 2019). Other brain structures exhibiting analogous patch-matrix-like neurochemical blueprints (e.g., striatum: Gerfen, 1985, 1992; Brimblecombe and Cragg, 2017) are known to rely upon Eph-ephrin signaling for cell sorting within developing structural compartments (Passante et al., 2008), as well as guiding the specificity of interfacing afferent-efferent streams (Tai and Kromer, 2014). In the striatum, spatially distinct Eph-ephrin guidance patterns delineate aspects of its emerging compartmental architecture (Janis et al., 1999), as well as discrete sub-compartmental zones receiving highly localized innervation schemes (Tai et al., 2013; Tai and Kromer, 2014). EphA4 and EphB1 are preferentially expressed in the matrix during an early postnatal critical period before subsequent downregulation (Martone et al., 1997; Richards et al., 2007; Passante et al., 2008; Tai et al., 2013). Although also temporally regulated, EphA7 exhibits a “matrisome-like” pattern, aligning with aspects of the matrix in the dorsal striatum, while partially overlapping striosomes or patches in the ventral striatum (Tai et al., 2013; Tai and Kromer, 2014). To date, no examined Eph-ephrin members exhibit expression highlighting the entirety of its characteristic μ-opioid receptor-positive striosomes.

The LCIC shares many similarities with the described developmental progression of the striatum, namely the early emergence of its compartmental organization and segregated circuitry. While precise mechanistic roles require further study, it appears Eph-ephrin interactions may play similarly significant LCIC roles in regards to neuronal sorting (e.g., into modules vs matrix) and axonal targeting (modular vs matrix-specific inputs). Previous reports from our lab identified EphA4 and ephrin-B2 as having discrete LCIC expression patterns that align with GAD-positive modular fields and are offset from the CR-positive matrix (Gabriele et al., 2011; Wallace et al., 2016; Gay et al., 2018). Such expression is transient, peaking during the early postnatal period as LCIC compartments emerge (Dillingham et al., 2017) and afferent projection distributions sharpen (Lamb-Echegaray et al., 2019). *In situ* hybridization data in developing mouse (Allen Brain Atlas^1^, *see the summary figure* in Wallace et al., 2016) suggest additional Eph-ephrin members likely influence the emergence of orderly IC networks.

Here, we show a similar temporal regulation for ephrin-B3 that exhibits a pattern complementary to that described previously for EphA4 and ephrin-B2 (Gabriele et al., 2011; Wallace et al., 2016; Gay et al., 2018). Instead of aligning with LCIC modular fields, the ephrin-B3 expression is restricted to the encompassing CR-positive extramodular matrix. Despite sharing many common organizational features, namely the same early critical period (P0–P12) and transient expression of discrete Eph-ephrin patterns, both the LCIC and striatum exhibit unique developmental differences. For one, all LCIC Eph-ephrin expression patterns examined thus far strictly adhere to its neurochemically-defined compartmental boundaries. Each is either distinctly modular or extramodular in their expression, and thus either overlap or are complementary to their counterparts. It remains to be determined if other members have more restricted expression within the modules or surrounding matrix, or perhaps partially overlap aspects of both as is the case for EphA7 in the striatum. Topographic mapping of corticostriatal inputs arising from regions of S1 expressing high levels of ephrin-A5 selectively avoid EphA7-positive “matriosomes” (Tai and Kromer, 2014). In the LCIC, EphA4 modular expression consists of a dense, punctate fibrous network (Gay et al., 2018), in contrast to largely cellular ephrin-B2 (modular) and ephrin-B3 (matrix) labeling. Despite belonging to different Eph-ephrin subclasses (A and B), EphA4 is known to exhibit strong binding affinities for both ephrin-B2 and -B3 in addition to ephrin-A ligands (Wilkinson, 2001). The source(s) of the discontinuous EphA4-positive LCIC fibrous network remains to be determined. One might hypothesize they arise from sources of somatosensory origin and preferentially target modular zones *via* a series of complex interactions with resident LCIC neurons that occupy distinct compartments and exhibit unique Eph-ephrin molecular signatures. Ongoing studies in a variety of transgenic lines aim to pinpoint the precise mechanisms (e.g., permissive or repulsive, forward/reverse/bi-directional) whereby Eph-ephrin signaling instructs early multimodal LCIC projection specificity.

The discrete and complementary nature of LCIC guidance patterns directly contrasts that described previously in the neighboring central nucleus of the IC (CNIC). Instead of discrete zones of labeling, Eph-ephrins are expressed in gradients along its tonotopic axis and are important for accurate targeting and refinement of frequency-specific inputs (Gabriele et al., 2011; Wallace et al., 2013, 2016; Cramer and Gabriele, 2014). Despite dissimilar expression patterns and mapping features, it appears as if their critical periods for circuit assembly (Fathke and Gabriele, 2009; Wallace et al., 2013; Lamb-Echegaray et al., 2019) and the guidance proteins they utilize largely overlap. In both cases, topographic shaping coincides with peak Eph-ephrin expression (P0–P8) before subsequent downregulation once an early projection specificity is established. While the ephrin-B3 expression is negligible in the CNIC over this period, EphA4 and ephrin-B2 appear to play prominent roles in both subdivisions, given that their graded and discrete patterns correlate closely with the underlying microarchitecture (tonotopic layering vs. modular-extramodular compartments). This unique juxtaposition of two different types of emerging neural maps (CNIC: unimodal and continuous; LCIC: multimodal and discrete) within a given brain structure make the IC an intriguing model system for continued study. Understanding the mechanisms governing IC critical periods and the assembly of its varied topographic maps are necessary first-steps for determining how guidance errors or unrefined configurations might impact behavior and perhaps underlie certain neurodevelopmental conditions.

## Data Availability Statement

The raw data supporting the conclusions of this article will be made available by the authors, without undue reservation.

## Ethics Statement

The animal study was reviewed and approved by Institutional Animal Care and Use Committee of James Madison University (approval number, 20-1421). Written informed consent was obtained from the owners for the participation of their animals in this study.

## Author Contributions

JS and MG designed the experiments. JS executed all experimentation and associated imaging. CB and JC performed sampling for quantitative assessments. MG analyzed and interpreted the data, prepared all figures/tables, and wrote the article. All authors contributed to the article and approved the submitted version.

## Conflict of Interest

The authors declare that the research was conducted in the absence of any commercial or financial relationships that could be construed as a potential conflict of interest.

## Footnotes

^1^http://portal.brain-map.org
