# Supplementary material for: Registry of Compartmental Ephrin-B3 Guidance Patterns With Respect to Emerging Multimodal Midbrain Maps
Source: Front Neuroanat. 2021 Mar 16;15:649478. doi: 10.3389/fnana.2021.649478 (PMC8010652; doi:10.3389/fnana.2021.649478)
Supplement: Supplementary file 1 [file Data_Sheet_1.PDF]

TABLE 1. Antibody Information

Antibody name	Structure of immunogen	Manufacturer info.	Concentration used
Anti-calretinin	Recombinant human calretinin containing a 6-his tag at the N-terminal	Swant, CR 7697, RRID: AB_2619710, rabbit, polyclonal	1:250
Anti-ephrin-B3	*S. frugiperda* insect ovarian cell line *Sf* 21-derived recombinant human ephrin-B3; Leu28-Ser224; Accession # Q15768	R&D Systems, AF395, RRID:AB_2095814, goat, polyclonal	1:200
Alexa Fluor 350 donkey anti-rabbit IgG	IgG recognizes both heavy and light chains from rabbit	Thermo Fisher Scientific, A10039, RRID:AB_2534015, donkey	1:25
Biotinylated horse anti-goat IgG	IgG recognizes both heavy and light chains from goat	Vector Laboratories, BA-9500, RRID:AB_2336123, horse	1:600
